# Breast Cancer Patient Prognosis Is Determined by the Interplay between *TP53* Mutation and Alternative Transcript Expression: Insights from *TP53* Long Amplicon Digital PCR Assays

**DOI:** 10.3390/cancers13071531

**Published:** 2021-03-26

**Authors:** Annette Lasham, Nicholas Knowlton, Sunali Y. Mehta, Antony W. Braithwaite, Cristin G. Print

**Affiliations:** 1 Department of Molecular Medicine and Pathology, School of Medical Sciences, University of Auckland, Auckland 1142, New Zealand; n.knowlton@auckland.ac.nz (N.K.); c.print@auckland.ac.nz (C.G.P.); 2 Maurice Wilkins Centre, University of Auckland, Auckland 1010, New Zealand; sunali.mehta@otago.ac.nz (S.Y.M.); antony.braithwaite@otago.ac.nz (A.W.B.); 3 Department of Pathology, University of Otago, Dunedin 9016, New Zealand; 4 Malaghan Institute of Medical Research, Wellington 6242, New Zealand

**Keywords:** *TP53* isoforms, alternative splicing, breast cancer prognosis, long amplicon digital PCR

## Abstract

**Simple Summary:**

The *TP53* gene, the most commonly mutated gene in human cancers, is capable of producing multiple RNAs (transcripts). The aim of our study was to measure the abundance of each *TP53* transcript, combined with *TP53* gene mutation information, to determine the interplay between these in a cohort of breast tumors from New Zealand patients. To do this, we devised a new assay which then enabled the measurement of all known *TP53* transcripts. We showed how *TP53* gene mutations influenced the levels of specific *TP53* transcripts in breast tumors. We evaluated whether a combination of *TP53* tumor information, including *TP53* mutation status and the levels of certain *TP53* transcripts, with standard clinical and pathological information, was associated with breast cancer patient outcome. We recommend that a truly comprehensive analysis of *TP53* needs to incorporate data about both *TP53* DNA mutations and the expression of the alternative *TP53* transcripts.

**Abstract:**

The *TP53* gene locus is capable of producing multiple RNA transcripts encoding the different p53 protein isoforms. We recently described multiplex long amplicon droplet digital PCR (ddPCR) assays to quantify seven of eight *TP53* reference transcripts in human tumors. Here, we describe a new long amplicon ddPCR assay to quantify expression of the eighth *TP53* reference transcript encoding ∆40p53α. We then applied these assays, alongside DNA sequencing of the *TP53* gene locus, to tumors from a cohort of New Zealand (NZ) breast cancer patients. We found a high prevalence of mutations at *TP53* splice sites in the NZ breast cancer cohort. Mutations at *TP53* intron 4 splice sites were associated with overexpression of ∆*133TP53* transcripts. Cox proportional hazards survival analysis showed that interplay between *TP53* mutation status and expression of *TP53* transcript variants was significantly associated with patient outcome, over and above standard clinical and pathological information. In particular, patients with no *TP53* mutation and a low ratio of *TP53* transcripts *t2* to *t1*, which derive from alternative intron 1 acceptor splice sites, had a remarkably good outcome. We suggest that this type of analysis, integrating mutation and transcript expression, provides a step-change in our understanding of *TP53* in cancer.

## 1. Introduction

**Figure 1:**
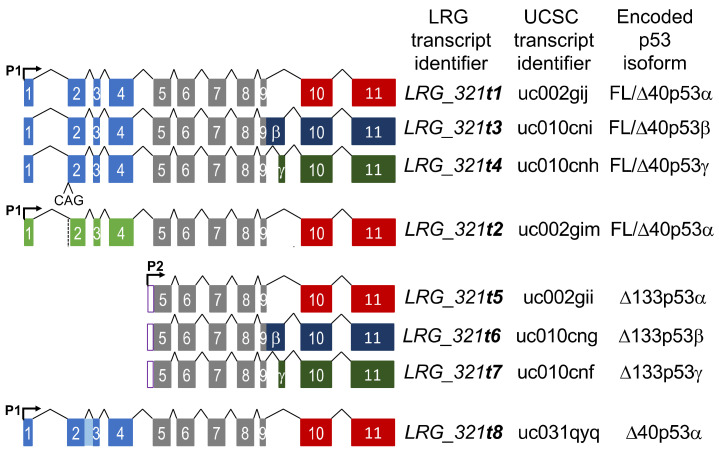
Schematic showing the structure of the eight *TP53* reference transcripts. The Locus Reference Genome (LRG) transcript identifier, the UCSC transcript identifier and the predicted encoded p53 isoforms are also shown for each *TP53* transcript. P1 and P2 refer to the P1 and P2 promoters, respectively. The light blue and light green boxes refer to transcripts with or without an additional CAG at the beginning of exon 2, respectively. The shaded light blue box between exons 2 and 3 is retention of intron 2 within the ∆40p53-encoding transcript, *t8*. The grey boxes indicate the exons common to all transcripts. The red, dark blue and dark green boxes refer to transcripts encoding α, β and γ C-terminal ends.

The tumor suppressor protein p53, encoded by the *TP53* gene, is the most commonly mutated gene in human cancers [1]. In international breast cancer datasets, somatic *TP53* mutations have been reported to occur in 22.8–34% of cases [2,3,4]. *TP53* is the most frequently mutated gene in more aggressive breast cancer subtypes, the estrogen receptor (ER) negative Her2+ and triple negative (TNBC) breast cancers, where *TP53* mutations occur in more than 50% of cases [3,4,5,6]. A recent integrative analysis of the role of *TP53* in over 10,000 patient samples from 32 cancer types, combining *TP53* copy number, mutation status and whole *TP53* gene locus-level RNA-seq data showed that *TP53* RNA abundance was influenced by the presence and type of *TP53* mutation [4]. These findings are in accord with our previous demonstration in breast cancer that specific classes of *TP53* mutation are associated with enhanced or reduced *TP53* locus expression [7]. The *TP53* gene locus is capable of expressing multiple RNA transcripts, generated by alternative gene promoter usage and alternative RNA splicing, which encode multiple p53 protein isoforms (Figure 1; [8]).

The Ensembl locus reference genomic record for *TP53* (*LRG_321*) describes eight reference transcripts, *t1*–*t8* [9,10], although more are predicted [11]. All *TP53* transcripts have a common region of 618 bp, spanning exons 5–9. Until recently, the length of this common region prevented the end-to-end detection required for precise quantitation of the individual RNA transcripts by standard RT-qPCR or short-read RNA-sequencing [12,13]. Therefore, what is currently known of the range of *TP53* transcripts has focused on quantitation of either the 5′ or 3′ end sequences, predominantly using RT-qPCR [11]. This approach has shown that the levels of the isoform “ends” are associated with various clinical or pathological features of breast cancer. For example, elevated levels of ∆*40TP53* 5′ ends have been observed in breast compared to normal tissues [14]. The different *TP53* RNA transcripts play distinct biological roles and in breast cancer, the *TP53β* 3′ end levels have been negatively associated with tumor size, and positively associated with estrogen receptor (ER) positive tumors and better patient outcomes [14,15]. Higher expression of the *TP53γ* 3′ end in breast cancers with a mutant p53 has been associated with good patient prognosis [15]. Using nested PCR to detect the transcript encoding the ∆133p53β isoform, breast cancer patients with the highest levels had the poorest prognosis [16].

In the accompanying manuscript, we describe multiplex long amplicon ddPCR assays to quantitate seven of the individual *TP53* reference transcripts in cell lines and fresh-frozen breast tumors [13]. We showed that five of these *TP53* reference transcripts were co-expressed in all breast tumors analyzed and that non-canonical *TP53* transcripts could also be identified [13]. Depending on their position, *TP53* DNA mutations will be transcribed into some but not all *TP53* RNA transcripts, altering the biological function of only that subset of p53 protein isoforms that incorporate the translated mutations. In addition, *TP53* DNA mutations may alter splicing patterns and influence the relative abundance of the various alternative *TP53* RNAs and their translated protein isoforms. Therefore, a truly comprehensive analysis of *TP53* needs to incorporate data about both *TP53* DNA mutations and the expression of the alternative *TP53* RNA transcripts.

In this study, we combine gene sequencing with long amplicon ddPCR assays to integrate information about *TP53* transcript abundance, *TP53* mutation status and clinical and pathological information in cohort of tumors from 89 breast cancer patients. Our analysis includes a new multiplex long amplicon ddPCR assay to detect the final *TP53* reference transcript, *LRG_321t8*, encoding ∆40p53α, thereby providing information about all eight *TP53* reference transcripts. We observe a high number of *TP53* splice site mutations in this breast cancer cohort, which we find influence *TP53* transcript variant expression. We also show that information about *TP53* mutations and the abundance of alternatively spliced *TP53* RNA transcripts is associated with patient outcome and strengthens prognostic associations when added to standard clinical and pathological information.

## 2. Results

### 2.1. A Cohort of New Zealand Breast Cancers Has a High Proportion of TP53 Splicing Mutations

**Figure 2:**
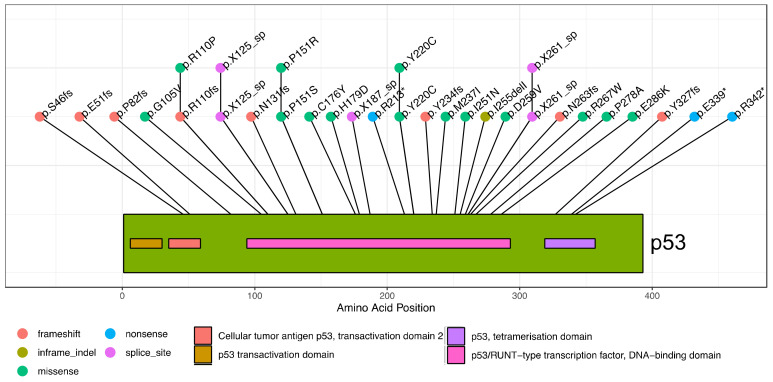
Lollipop plot showing location and sequence of *TP53* mutations in breast cancer cohort. The individual mutations are indicated with their amino acid change given. Mutations preceded with “X” are splice site mutations, located within introns. The color of the lollipop indicates the mutation type. A schematic of the p53 protein is shown, with domains indicated in the color key below the plot.

We mapped somatic *TP53* mutations in breast tumors of 89 New Zealand patients using targeted sequencing of the *TP53* gene locus. This identified 31 tumors with mutations that influence the *TP53* coding region, accounting for ~35% of the cohort (Table S1). These mutations occurred along the length of the *TP53* gene, with 45% being missense (Table 1 and Figure 2).

While we did not observe some *TP53* hotspot mutations commonly reported in international breast cancer studies such as: R175, R248, R273, we did observe many frequent mutations including R110, H179, R213*, Y220C, R342* [3,6,17,18,19].

### 2.2. Detailed Analysis of TP53 Splicing Mutations and Their Consequences on RNA Transcript Expression

**Figure 3:**
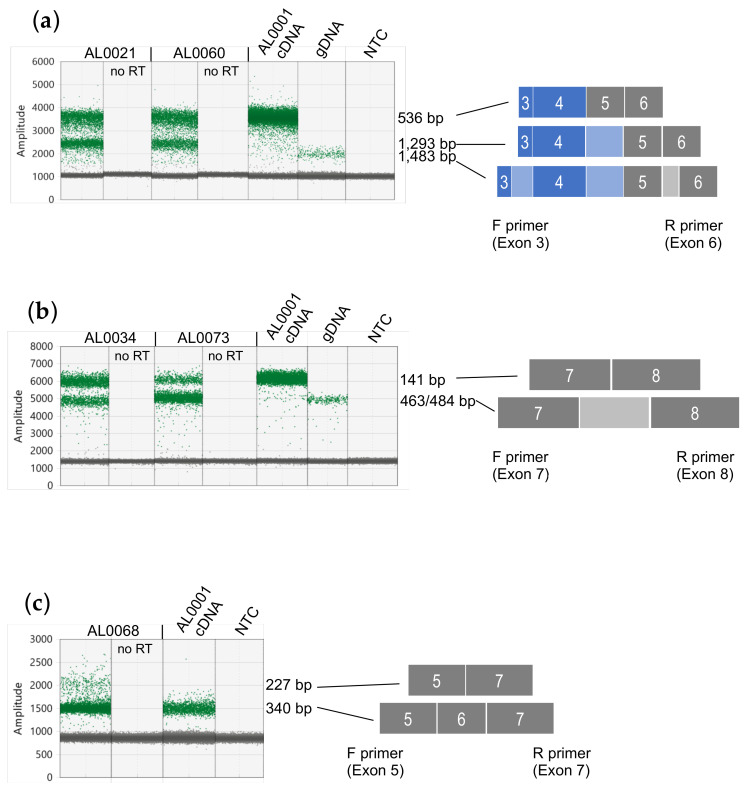
Validation of *TP53* splicing mutations in breast cancer samples. (**a**) ddPCR assay between *TP53* exons 3 and 6 showing amplification of *TP53* transcripts. AL0021 and AL0060 have a point mutation in the *TP53* intron 4 donor splice site at +1 and +5, respectively. AL0001 has no splicing mutations and was used as a control to show the fluorescence amplitude of a correctly spliced *TP53* RNA (536 bp), whereas those *TP53* RNAs retaining intron 4 have an amplicon size of 1293 bp. Genomic DNA (gDNA) was used as a control template to show an amplicon with retention of introns 3, 4 and 5. (**b**) ddPCR assay between *TP53* exons 7 and 8 showing amplification of *TP53* transcripts. AL0034 and AL0073 have a 21 bp deletion spanning the *TP53* intron 7 donor splice site or a point mutation at +1 in the *TP53* intron 7 donor splice site, respectively. AL0001 has no splicing mutations and was used as a control to show the fluorescence amplitude of a correctly spliced *TP53* RNA (141 bp), whereas those *TP53* RNAs retaining intron 7 have an amplicon size of 463 or 484 bp for AL0034 and AL0073, respectively. Genomic DNA (gDNA) was used as a control template to show an amplicon with retention of intron 7 (484 bp). (**c**) ddPCR assay between *TP53* exons 5 and 7 showing amplification of *TP53* transcripts. AL0068 has a point mutation at −1 in the *TP53* intron 5 acceptor splice site. AL0001 has no splicing mutations and was used as a control to show the fluorescence amplitude of a correctly spliced *TP53* RNA (340 bp), whereas those *TP53* RNAs skipping exon 6 have an amplicon size of 227 bp. ddPCR results shown are “1-D” plots, with green dots representing droplets where PCR products have been amplified and grey dots represent no amplified PCR product. The fluorescence amplitude (Amplitude) on the y-axes is indicative of amplicon size/s within each assay.

We noted a moderate proportion of mutations predicted to influence *TP53* transcript splicing (16%). These include mutations within intron 4, intron 5 and intron 7 (Table S1). To confirm and quantitate the *TP53* transcripts expressed in tumors with these mutations, we performed long amplicon ddPCR assays between specific exons using cDNA prepared from RNA as template to confirm that all *TP53* splice mutations were expressed. For tumors with intron 4 splice site mutations, AL0021 and AL0060, these both had mutations within the donor splice site, at +1 and +5, respectively. Although an additional transcript retaining the 757 bp intron 4 was clearly evident in the *t1* long amplicon ddPCR assays, there was insufficient separation between the two amplified products to accurately quantitate both transcripts [13]. Therefore, we designed specific ddPCR assays to amplify a region of *TP53* RNA between exons 3 and 6, which generated a smaller amplicon able to clearly resolve and quantitate *TP53* RNAs retaining intron 4. This confirmed that both the AL0021 and AL0060 tumors expressed two *TP53* transcripts, with and without retention of intron 4 (amplicon sizes 1,293 bp and 536 bp, respectively, Figure 3a). cDNA from tumor AL0001 was used as a control to show amplification of canonically spliced *TP53* RNA, and genomic DNA to demonstrate the amplicon size when *TP53* introns 3, 4 and 5 are retained (amplicon size of 1,483 bp). From the *TP53* gene sequencing, the mutant allele frequency was 58% and 53% (Table S1), and quantitation of the products of these ddPCR assays showed that the alternative (intron 4-retaining) transcript comprised 49% and 40% of the *TP53* transcripts for tumors AL0021 and AL0060, respectively.

Tumors AL0034 and AL0073 had mutations that we predicted would affect the correct splicing of intron 7. AL0034 had a 21 bp deletion that spanned 14 bases at the 3′ end of exon 7 and the first 7 bases in intron 7, and AL0073 had a point mutation at +1 in the intron donor splice site. To test this prediction, a ddPCR assay was designed to amplify *TP53* RNA between exons 7 and 8 to determine whether intron 7 was retained in some *TP53* transcripts. This confirmed that both AL0034 and AL0073 had *TP53* RNAs that retained intron 7 (amplicon size 463/484 bp, respectively), in addition to a canonically spliced RNA (amplicon size 141 bp; Figure 3b). cDNA from tumor AL0001 was used as a control to show amplification of spliced *TP53* RNA and genomic DNA to show when intron 7 was retained. From the *TP53* gene sequencing we had observed that the mutant allele frequency was 32% and 39% for AL0034 and AL0073, respectively. From quantitation of the individual ddPCR products in this assay, the alternative (intron 7-retaining) RNAs comprised 36% and 82% of the *TP53* RNAs for AL0034 and AL0073, respectively (Figure 3b).

Tumor AL0068 had a low allele frequency splicing mutation (13%) at −1 of the acceptor splice site of intron 5. This would also be present in all *TP53* reference transcripts. A ddPCR assay designed between exons 5 and 7 showed that a *TP53* RNA smaller than the expected size was also amplified, consistent with this mutation causing omission of exon 6, but not retention of intron 5 (Figure 3c). Quantitation of the individual ddPCR products in this assay showed that RNAs skipping *TP53* exon 6 comprised 5% of the total *TP53* transcripts in tumor AL0068. Tumor AL0001 was used as a control to show amplification of spliced *TP53* RNA (amplicon size of 340 bp).

### 2.3. TP53 Intron 4 Splice Mutations Are a Mechanism to Overexpress ∆133TP53 Transcripts

**Figure 4:**
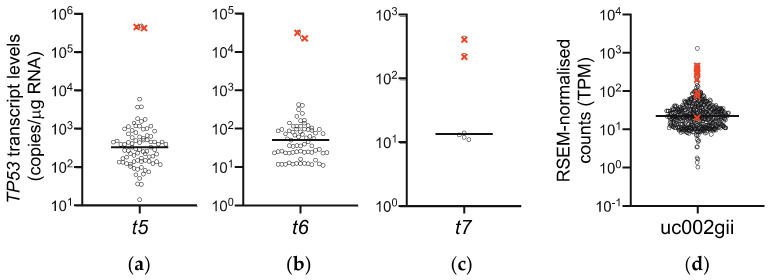
Scatterplots showing levels of transcripts encoding ∆133p53 isoforms in Breast Cancer cohorts. (**a**–**c**) Results from long amplicon ddPCR of 85 NZ breast cancer samples with both known *TP53* mutation status and *TP53* transcript expression data. (**a**) *LRG_321t5* encoding ∆133p53α (detectable in 83/85 tumors), (**b**) *LRG_321t6* encoding ∆133p53β (detectable in 73/85 tumors), (**c**) *LRG_321t7* encoding ∆133p53γ (detectable in 6/85 tumors) and (**d**) uc002gii *TP53* transcript, assigned as encoding ∆133p53α, quantitated by RSEM analysis of RNA-seq data in 727 TCGA BRCA samples with known *TP53* mutation status (TPM = transcripts per million reads). Black circles represent tumor samples expressing this transcript; for clarity samples have been jittered on the *x*-axis. Tumors with a *TP53* intron 4 splice site mutation are indicated by **×**. Horizontal line represents the median expression level for each cohort. Note that “detectable” refers to limit of detection of ddPCR assays, which is transcripts with abundance greater than 10 copies/μg RNA.

Using our long amplicon ddPCR assays to quantitate the individual *TP53* transcripts [13], we observed that both tumors with the intron 4 splicing mutation (AL0021 and AL0060) expressed extremely high levels of the *t5* transcript, encoding the ∆133p53α isoform, compared to samples in the rest of the cohort (Figure 4a).

These tumors also expressed extremely high levels of the *t6* and *t7* transcripts (Figure 4b,c; for transcript positions refer to Figure 1). In order to determine whether this was a feature of *TP53* intron 4 splice mutations, we examined RNA-seq data from the TCGA BRCA cohort [20,21]. 727 samples that had both *TP53* mutation status reported and expressed the transcript assigned to encode ∆133p53α (transcript uc002gii, referred to as *t5* here; see Figure 1), were analyzed. Eight BRCA tumors were reported to have a *TP53* intron 4 splice site mutation, and consistent with our results, seven of these expressed the uc002gii transcript at ≥95th percentile (Figure 4d). Transcripts assigned as encoding ∆133p53β (uc010cng, referred to as *t6* here) and ∆133p53γ (uc010cnf, referred to as *t7* here) were only measurable by RNA-seq in 10% and 1.8% of samples, respectively, so were not included in this analysis. Since intron 4 contains the 5′ untranslated region of the ∆*133TP53* transcripts, these transcripts are not *t5*–*t7* per se (i.e., driven off the P2 promoter), but are *t1*, *t4* and *t5* transcripts retaining intron 4, allowing the P1 promoter to drive expression of transcripts that have the *t5*–*t7*-specific 5′ end sequences. This suggests that *TP53* intron 4 splice mutations may provide a mechanism to overexpress transcripts encoding the ∆133p53 isoforms.

### 2.4. Analysis of All TP53 Reference Transcripts in New Zealand Breast Cancer Cohort

Our previous work described the long amplicon ddPCR assays that were used to quantitate the expression of seven *TP53* transcripts encoding FL/∆40p53α, β and γ, and ∆133p53α, β and γ (*LRG_321* transcripts *t1*–*t7*; see Figure 1) in a New Zealand breast cancer cohort [13]. Building on this work and using an additional ddPCR assay to quantitate *TP53* reference transcript *LRG_321t8* that encodes the ∆40p53α isoform, we aimed to determine whether the relative abundance of the eight individual *TP53* reference transcripts and the mutations they carry were associated with any clinical or pathological features.

#### 2.4.1. Quantitation of ∆40p53-Encoding Transcripts

The new assay to quantitate reference transcript *LRG_321t8* differed from the previous designs by the forward primer being located with intron 2 [22]. This allowed detection and quantitation of the *t8* transcript, encoding ∆40p53α, and also any β or γ-encoding ∆40p53 transcripts (Figure S1a). In the New Zealand breast cancer cohort we found that the *t8* transcript was detectable in all tumors (from 67 to 17,169 copies/μg RNA), with a ∆40p53β-encoding transcript detectable in 51 tumors (referred to as *t8β*; from 32 to 714 copies/μg RNA) and a ∆40p53γ-encoding transcript detectable in only 2 tumors (referred to as *t8*γ; 33 and 35 copies/μg RNA) (Figure S1b). Plotting the abundance of all *TP53* transcripts in this cohort showed that the *t8* transcript was of similar abundance to the *TP53 t2* and *t3* transcripts (encoding FL/∆40p53α and FL/∆40p53β, respectively; Figure S1b).

#### 2.4.2. Association of *TP53* Mutation Type with Expression of Individual *TP53* RNA Transcripts

**Figure 5:**
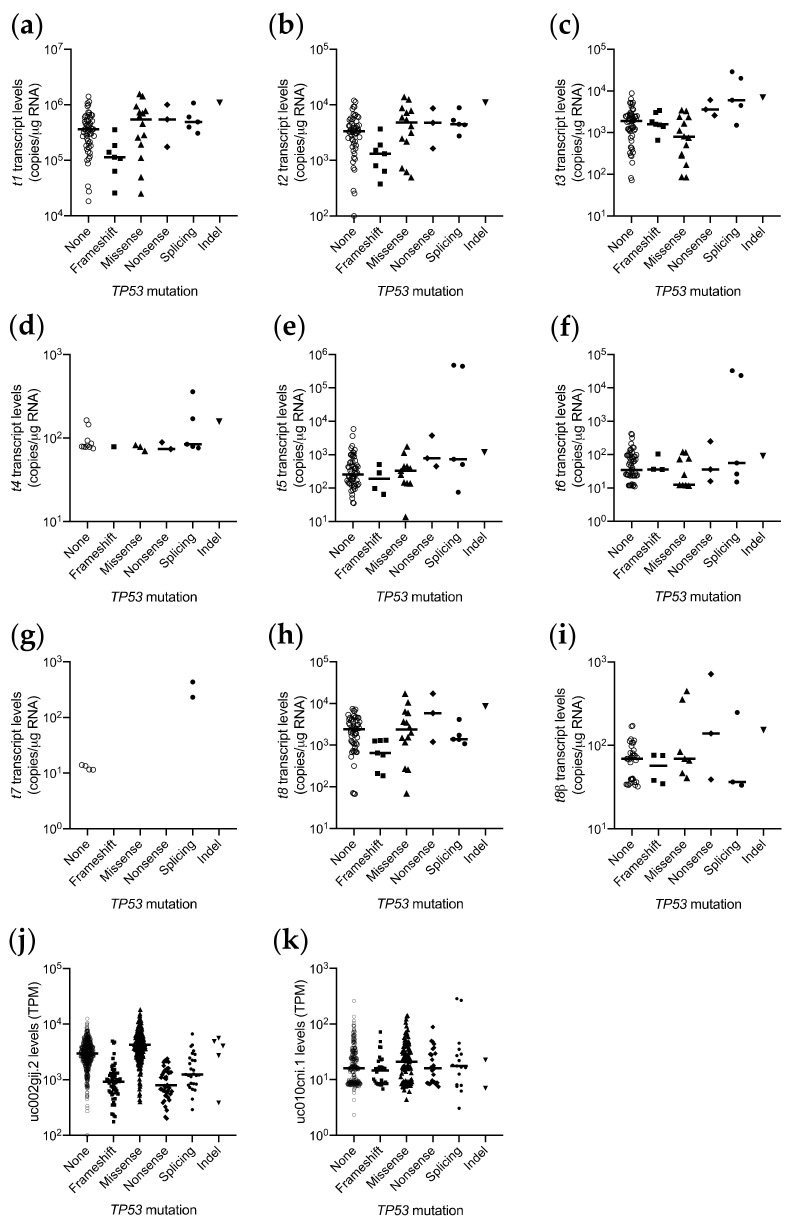
Scatterplots showing *TP53* transcript abundance by *TP53* mutation status in breast cancer cohorts. (**a**–**i**) *TP53* transcript levels in New Zealand breast cancer cohort, quantitated by long amplicon ddPCR. (**a**) *LRG_321t1* encoding FL/∆40p53α, (**b**) *LRG_321t2* encoding FL/∆40p53α, (**c**) *LRG_321t3* encoding FL/∆40p53β, (**d**) *LRG_321t4* encoding FL/∆40p53γ, (**e**) *LRG_321t5* encoding ∆133p53α, (**f**) *LRG_321t6* encoding ∆133p53β, (**g**) *LRG_321t7* encoding ∆133p53γ, (**h**) *LRG_321t8* encoding ∆40p53α, and (**i**) *t8β* encoding ∆40p53β, in each tumor. (**j**,**k**) *TP53* transcript levels from the TCGA BRCA cohort, quantitated by RNA-seq, (**j**) uc002gij.2, encoding FL/∆40p53α, (**k**) uc010cni.1, encoding FL/∆40p53β. Bar represents the median, with symbols representing individual tumor samples.

We next examined the relationship between *TP53* mutation type and expression levels of individual *TP53* RNA transcripts. (Figure 5a–i). In our cohort, 85/89 breast tumors had both *TP53* transcript and *TP53* mutation information. Although the numbers were small, analysis of abundance of transcripts encoding FL or ∆40p53 isoforms with an α C-terminal end (transcripts *t1*, *t2*, *t8*) showed that those with a frameshift mutation were present at lower levels than those α-encoding transcripts with a missense or no *TP53* mutation (e.g., for *t1* transcripts encoding FL/∆40*TP53*α, *p* = 0.017, Figure 5a), concordant with our previous finding analyzing the whole *TP53* gene locus-level expression [7]. However, we did not observe this for transcripts encoding FLp53 or ∆40p53 isoforms with a β C-terminal end (transcripts *t3* and *t8β*), where there was no association between mutation type and RNA abundance (for *t3* transcripts encoding FL/∆40p53β, *p* = ns, Figure 5c). We also saw no association between mutation type and RNA abundance for the *t5* and *t6* transcripts (encoding ∆133p53α and β, respectively), however only four and three frameshift mutations would be present in the ∆133p53α and β sequences of these tumors, respectively (Figure 5e,f). To determine whether this observation with transcripts encoding the FL/∆40p53α and β isoforms was also seen in another cohort, we analyzed RNA-seq data from the TCGA BRCA cohort, by plotting transcript levels of uc002gij.2 and uc010cni.1 (assigned as encoding FL/∆40p53α and FL/∆40p53β, respectively) by *TP53* mutation type. These results were consistent with the findings from our cohort, that the transcripts encoding the FL/∆40p53α protein with *TP53* frameshift mutations were less abundant than those with missense or no *TP53* mutations (*p* < 0.0001, Figure 5j). However, the abundance of transcripts encoding FL/∆40p53β protein were not significantly different by *TP53* mutation type or compared to those with no *TP53* mutation (*p* = ns, Figure 5k).

We also noted that the expression of the *t4* transcript (encoding FL/∆40p53γ) was associated with *TP53* mutation status. Although only 22 of 85 tumors had detectable levels of the *t4* transcript, 12 of these were tumors with *TP53* mutations (*p* = 0.048). Interestingly, all five tumors with a *TP53* splicing mutation had detectable levels of the *t4* transcript (Figure 5d).

### 2.5. TP53 Information Is Associated with Clinical and Pathological Features

We then determined whether the levels of the transcripts encoding the p53 isoforms were associated with intrinsic breast cancer subtype [23]. Using molecular subtype information generated for these tumors [24], the levels of the individual transcripts were plotted by individual subtype. This showed that in our cohort, the levels of each *TP53* transcript were similar across all subtypes (Figure S2; Table S2). Only *t8* showed a modest association with subtype (*p* = 0.042), but was not significant after false discovery rate control. These results suggest that the expression of the individual *TP53* transcripts is intrinsic to all breast cancers and is not related to breast cancer subtype.

Next we determined whether the levels of the transcripts encoding the p53 isoforms showed any nominal univariate associations with any clinical or pathological features of breast cancer. We used two approaches; either using the concentration of the individual *TP53* transcripts (i.e., copies of each transcript/μg tumor RNA) or the ratio of each of the individual *TP53* transcripts to the *t1* transcript (encoding FL/∆40p53α), as this was the most abundant transcript, and since the use of relative transcript levels provides an internal control for sample-specific factors such as differences in efficiency of cDNA synthesis between tumor samples. We also quantitated the *TP53* 5′ and 3′ ends as previously [12] and included these in the analysis. The most significant univariate associations were the *t3*/*t1* ratio with tumor ER status (*p* = 4.7 × 10^−4^), PgR status (*p* = 1.2 × 10^−3^) and breast cancer subtype (*p* = 9.8 × 10^−5^) (Table S2).

We then determined whether other *TP53* information, such as *TP53* gene mutation status or likely loss of the wild type *TP53* allele (LOH; loss of heterozygosity), was associated with any clinical or pathological features. This showed that the *TP53* mutation status was strongly associated with breast cancer subtype (*p* = 4.7 × 10^−5^), tumor ER (*p* = 1.3 × 10^−4^) and PgR status (*p* = 6.5 × 10^−6^) and histological grade (*p* = 4.4 × 10^−6^) (Table S2). However, *TP53* LOH was not associated with any clinicopathological features in this cohort.

### 2.6. TP53 Mutation Status and t2/t1 Transcript Ratio Are Associated with Breast Cancer Patient Outcome

**Figure 6:**
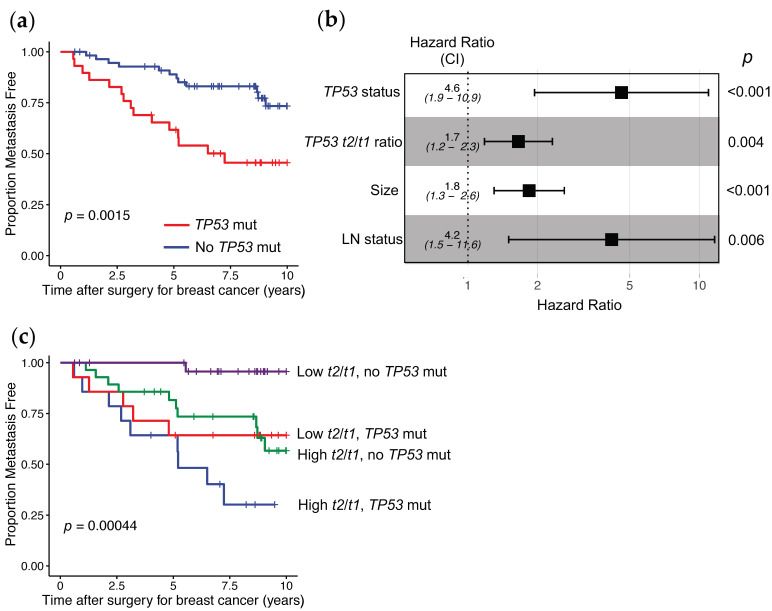
Association of *TP53* tumor information with distant metastases free survival in NZ breast cancer patients. (**a**) Kaplan–Meier curves showing the proportion of patients having a distant metastatic event by their *TP53* tumor mutation status (log rank test *p* = 0.0015). Red line = patients with tumors with a *TP53* mutation (*n* = 31), Blue line = patients with tumors with no *TP53* mutation (*n* = 58). (**b**) Forest plot showing contribution of *TP53* tumor information and clinicopathological features to a multivariable Cox proportional hazards model predicting those patients having a distant metastatic event (*n* = 83 with 27 patients developing distant metastases before 12 years, log rank test *p* = 4.2 × 10^−8^), CI = 95% confidence intervals, (**c**) Kaplan–Meier curves showing the proportion of patients having a distant metastatic event by their *TP53* tumor mutation status and *t2*/*t1* transcript level ratios to stratify patients into four groups (log rank test *p* = 4.4 × 10^−4^). “High” = greater than median and “Low” = less than median levels of *t2*/*t1*, Green line = patients with tumors with High *t2*/*t1* levels and no *TP53* mutation (*n* = 27), Blue line = patients with tumors with High *t2*/*t1* levels and a *TP53* mutation (*n* = 16), Purple line = patients with tumors with Low *t2*/*t1* levels and no *TP53* mutation (*n* = 28) and Red line = patients with tumors with Low *t2*/*t1* levels and a *TP53* mutation (*n* = 14).

We then evaluated whether the levels of the individual *TP53* transcripts and/or the *TP53* mutational status were associated with patient prognosis. We observed that *TP53* mutational status was associated with prognosis, as patients having tumors with a *TP53* mutation were more likely to have a distant metastatic event than those with no *TP53* mutation (Figure 6a, *p* = 1.5 × 10^−3^).

Next we used Cox proportional hazard regression models to determine whether there was any relationship between the various types of *TP53* information and patient outcome. For the individual *TP53* transcripts, we applied two approaches to evaluate how these were associated with prognosis; either using the concentration of the individual transcripts (i.e., copies of each transcript/μg tumor RNA) or the ratio of each of the individual *TP53* transcripts to the *t1* transcript (encoding FL/∆40p53α), as this was the most abundant transcript. As univariate tumor markers of distant metastases free survival (DMFS), we observed that the *TP53* mutational status, the abundance of *TP53*α 3′ RNA end, the abundance of *TP53* transcript *t2* to *TP53* reference transcript *t1*, and the ratio of the ∆133*TP53* 5′ end to the FL/∆40*TP53*_*T1* 5′ end, were significantly associated with patient prognosis. However, after correcting for the false discovery rate, only the *TP53* mutation status and the abundance of *TP53* transcript *t2* to reference transcript *t1* had significant prognostic associations (hazard ratio (HR), 3.19; 95% confidence intervals (CI), 1.49–6.84; *p* = 2.9 × 10^−3^ for *TP53* mutation status and HR, 1.88; 95% CI, 1.38–2.56; *p* = 7.3 × 10^−5^ for *TP53* *t2*/*t1* ratio; Table 2). Then, the association of these two *TP53* tumor markers was analyzed further, by adjusting for patient lymph node status, tumor size, histological grade, and estrogen receptor (ER) and progesterone receptor (PgR) status. We found that the *TP53* mutation status or the *TP53* *t2*/*t1* ratio provided additional information independent of the clinicopathological features and were both significantly associated with DMFS in all patients (HR, 4.36; 95% CI, 1.47–12.97; *p* = 8.1 × 10^−3^ for the *TP53* mutation status; HR, 1.85; 95% CI, 1.32–2.59; *p* = 3.4 × 10^−4^ for *TP53* *t2*/*t1* ratio; Table 2).

As a number of risk prediction models for breast cancer patient prognosis have been developed from standard clinico-pathological markers [25,26,27], we wished to determine whether incorporation of this *TP53* information generated an improved prognostic model. Therefore, we performed multivariable analysis, combining clinical and pathological information (tumor ER and PgR status, size, and histological grade, and patient lymph node status) with *TP53* mutation status and *TP53* *t2* transcript abundance relative to the most abundant *t1* transcript. These results showed that, with the exception of lymph node status, and tumor size, the combined *TP53* genomic information contributed significantly in a Cox proportional hazards model to associate with patients’ DMFS, than the other clinicopathological markers (HR, 1.84; 95% CI, 1.30–2.61; *p* = 6.3 × 10^−4^ for tumor size; HR, 4.18; 95% CI, 1.50–11.64; *p* = 6.2 × 10^−3^ for lymph node status; HR, 4.61; 95% CI, 1.94–10.95; *p* = 5.4 × 10^−4^ for *TP53* mutation status; HR, 1.65; 95% CI, 1.18–2.32; *p* = 3.6 × 10^−3^ for *TP53* *t2*/*t1* ratio; Table 3.) These results are shown graphically in Figure 6b.

Multivariable Cox models are difficult to visualise, so we plotted a rough approximation using data split into binary categories in Kaplan–Meier plots. Specifically, the patients were stratified into four groups based on the *TP53* *t2*/*t1* ratio and also the *TP53* mutation status of their tumors. This showed that patients with a *TP53* tumor mutation, irrespective of the *t2*/*t1* ratio, or those with tumors with greater than the median *t2*/*t1* ratio with no *TP53* mutation had the poorest prognosis (Figure 6c). However, patients with tumors expressing less than the median level of *t2*/*t1* ratio and with no *TP53* mutation, had very good prognosis, and only one patient in this group developed metastatic disease (Figure 6c). The results from both Figure 6a,c demonstrate that even patients with no tumor *TP53* mutation can have poor prognosis, but incorporation of *TP53* transcript information can be used to further stratify these to identify a group of patients with very good outcomes.

As the levels of the *TP53*β and *TP53*γ 3′ends in breast tumors have been associated with patient prognosis [14,15], based on *TP53* mutation status, we also analyzed how the individual transcripts that would contribute the most to these *TP53* 3′ end measurements, that being transcripts *t3* (encoding FL/∆40p53β) and *t4* (encoding FL/∆40p53γ), respectively, were associated with distant metastasis-free survival (DMFS) of patients. Patients were stratified by their tumors having a *TP53* mutation or not, and then by the level of *t3* or *t4* transcripts. The Kaplan–Meier plots showed that patients whose tumors had a *TP53* mutation and less than median levels of the *t3* transcript, or those whose tumors had a *TP53* mutation and the *t4* transcript was undetectable, were more likely to have poorer prognosis than patients in the other groups (Figure S3). Patients with tumors expressing low *t3* transcript levels and with a *TP53* mutation had particularly poor prognosis (Figure S3a,c,e). In a small group of patients (*n* = 10) with no *TP53* tumor mutation but detectable levels of the *t4* transcript, none developed metastatic disease or died from breast cancer before 10 years (Figure S3d,f). However, as these effects had not been seen in a multivariable Cox model with interaction terms (above), this suggested that the associations observed are non-linear or apply to a subset of patients only.

## 3. Discussion

Here, we performed an integrated analysis of *TP53* mutation status and expression of the individual *TP53* transcripts in tumor samples donated by a cohort of New Zealand breast cancer patients. We also describe a novel long amplicon ddPCR assay to detect the final *TP53* reference transcript, *LRG_321t8*, which means that all eight individual *TP53* reference transcripts can be quantitated in cell lines and fresh frozen tumor samples. We use this information to provide new insights into the impact of individual *TP53* transcripts and *TP53* gene mutations, as well as their interplay, on breast cancer patient outcome.

Given that a recent report described frequent transcription of *TP53* DNA mutations into *TP53* RNA [28], for most tumors in this study we have assumed that the *TP53* DNA mutations will be transcribed into RNA, unless the mutations influence RNA stability. However, for a subset of tumors in this study (the five with *TP53* splicing mutations), we confirmed transcription into RNA. Although the proportion of breast tumors with a *TP53* mutation is consistent with international data, the proportion of these that are splice site mutations in the NZ cohort is considerably higher [2,3,4]. We observed that 16% of the *TP53* mutations would influence *TP53* transcript splicing, compared to 2% reported in public databases [4,19,29], and even a recent publication that showed that splice mutations accounted for 6.6% of *TP53* mutations in colorectal cancer [30]. One explanation for this may be that not all mutation callers are reporting mutations that will influence splicing. Other sites located around the splice junctions or within the intron may influence splicing, but are not reported as has been described [19,30,31]. In addition, in silico methods used in previous studies may underestimate the effects of mutations on splicing [32].

Our results suggest that *TP53* intron 4 splicing mutations have a significant effect on the *TP53* transcript profile/composition. Although others have described how truncated FL/∆40p53 isoforms would arise from these mutations [30,33], our data show that these effectively lead to very high levels of the *t5*–*t7* transcripts, driven off the *TP53* P1 promoter, and therefore predict that very high levels of the ∆133p53 isoforms would be expressed. A recent paper has highlighted the clinical relevance of *TP53* intron 4 splicing mutations. During platinum/paclitaxel-based neo-adjuvant treatment for high grade serous ovarian cancer, an intron 4 donor splice mutation (c.375 + 1G > A) became the highly proliferative major clone, suggesting that this *TP53* mutation was potentially associated with chemotherapy resistance [33]. A *TP53* mutation influencing the intron 4 donor splice site has also been reported in a Li-Fraumeni family [34].

Using long amplicon ddPCR methods also allowed us to examine how *TP53* splice site mutations manifest themselves in the transcripts expressed from the locus. In addition to those *TP53* mutations influencing the splicing of large introns, which are clearly visible in long amplicon ddPCR assays (such as retention of the 757 bp intron 4; [13]), another example was sample AL0073 where a splice site mutation in 39% of the *TP53* gene sequencing reads led to 82% of transcripts retaining intron 7, despite no evidence of LoH. As exons 5–9 are present in all *TP53* reference transcripts, retention of intron 7 would therefore impact all p53 isoforms expressed. For the intron 5 acceptor splice site mutation in sample AL0068, we saw no evidence of intron 5 retention, but instead the results suggested that exon 6 was skipped in a proportion of transcripts. This same mutation has been reported in colorectal cancers, where in the *TP53* RNA-seq data, exon 6 skipping was a more frequent event than intron 5 retention [30]. Our assays were sufficiently sensitive to detect intron 5 retention but did not, suggesting that exon 6 skipping is the predominant effect of this mutation.

We also showed that in addition to splice mutations, other *TP53* mutations also alter the proportion/composition of the *TP53* transcripts in the tumor. By analyzing the endogenous full length *TP53* transcripts, our results tentatively suggest frameshift mutations may affect the levels of the FL/∆40p53α- and FL/∆40p53β-encoding transcripts differently. Consistent with other studies [4,7,30], the abundance of transcripts encoding p53 isoforms with an α C-terminal end showed that those with a frameshift mutation were present at lower levels than those α-encoding transcripts with a missense or no *TP53* mutation. The explanation for this has been proposed to be that transcripts with frameshift mutations are subjected to nonsense-mediated decay (NMD) [4,30]. However, we observed that the *t3* transcript levels (encoding FL/∆40p53β) were similar irrespective of the type of *TP53* mutation. These findings suggest that endogenous *t3* transcripts with a frameshift mutation may be less susceptible to NMD. Therefore, certain *TP53* mutation types may not only alter the sequence but also the overall composition of the *TP53* isoforms expressed in a tumor. If the β-encoding *TP53* transcripts with frameshift mutations are not subjected to NMD, this is important because recent papers have shown that neo-open reading frames arise from transcripts with frameshift *TP53* mutations [35]. Therefore, the *t3* transcript may be a template for this phenomenon.

As shown by others [6,36], we observed that *TP53* mutational status was associated with prognosis, as patients having tumors with a *TP53* mutation were more likely to develop metastases than those with no *TP53* mutation. Our analysis of the associations of the individual *TP53* transcripts with breast cancer patient prognosis showed some results consistent with other studies reporting data from *TP53* 3′ end assays. For example, we observed that patients with a *TP53* tumor mutation and less than median (including undetectable) levels of transcripts *t3* and *t4* (encoding FL/∆40p53β and FL/∆40p53γ, respectively) had very poor prognosis, consistent with previous findings from analysis of the *TP53β* and *TP53γ* 3′ ends [14,15]. However, in contrast to previous studies [14,15], we did not observe that patients with tumors with high *t3* or detectable *t4* levels and *TP53* mutation had the best disease-free or breast cancer specific survival rates. In addition, detectable levels of the *t6* transcript encoding ∆133p53β by semi-quantitative PCR have been associated with breast cancer patient outcome [16], although analysis of *t6* transcript levels in our cohort was not associated with prognosis. Furthermore, we did not observe the association of these transcripts with prognosis in multivariable analysis across the entire cohort with or without interaction terms. This discrepancy (from analysis of patient subsets compared to analysis of all patients) indicates the association is less statistically robust and more likely due to cohort effects. There are several possible explanations for the inconsistencies between these results. Firstly, most studies have used RT-qPCR and we have used ddPCR. Furthermore, these previous studies have predominantly measured the *TP53* 3′ ends, which are providing information from multiple transcripts, whereas our assays are measuring individual transcripts. In addition, the NZ population is a distinctly unique group of ethnicities [37]; for *TP53* this is apparent given the proportion of *TP53* splicing mutations in our cohort being considerably higher than in international cohorts.

Significantly, we found that the ratio of the *t2*/*t1* transcripts and *TP53* mutation status of breast tumors were associated with distant metastases free survival of patients, independent of any clinical and pathological features. This *TP53* information also contributed more strongly than most clinical and pathological features, including tumor grade and ER status, to a model that predicted DMFS. The best multivariable Cox proportional hazards regression model (i.e., the model that explained the highest proportion of variance in patient outcome) was a combination of the ratio of the *t2*/*t1* transcripts and *TP53* mutation status of the breast tumors with tumor size and patient lymph node status. We also took an alternative approach to show how this *TP53* information was associated with DMFS, by stratifying patients into four groups based on their tumors having above or below *t2*/*t1* median levels and *TP53* mutation or not. This showed two groups that had very different outcomes, those patients with high *t2*/*t1* tumor levels and *TP53* mutation had frequent metastases, but a group of 28 patients (representing ~1/3 of the cohort) with low *t2*/*t1* tumor levels and no *TP53* mutation where only one patient developed metastases. Although studies have suggested that tumor *TP53* mutation status is not always a good indicator of prognosis [4,19], our data suggests that incorporating information from certain *TP53* transcripts, such as the *t2*/*t1* ratio, in addition to *TP53* mutation status of the tumor, may be a more accurate predictor of patient prognosis than clinical and pathological information alone.

The main limitation of our study is the small cohort size, therefore any conclusions may not be substantiated in studies of larger breast cancer cohorts. This is especially so in our analyses that stratified the cohort into four subgroups, as discussed above. Therefore, our findings need to be followed up in larger cohorts of breast cancer patient samples, exploiting the long amplicon ddPCR assays to quantitate the individual *TP53* transcripts in parallel to generating information about *TP53* mutation status and copy number, to provide a truly integrative perspective of the biological roles and clinical implications of *TP53* isoforms in breast cancer.

## 4. Materials and Methods

### 4.1. Patient Samples and Human Ethics

Breast cancer samples were a subset of our previous study [24], with informed written consent obtained from all study subjects and ethical approval granted by the New Zealand Multi-Region Ethics Committee (project MEC 09/06/060). Patient follow up data was obtained from the Breast Cancer Foundation NZ National Register. Clinical and pathological features of the patient samples used in this study are described in Table S3.

### 4.2. TP53 Gene Sequencing

Genomic DNA was prepared from the breast tumor samples described in [24], using Trizol (Thermo Fisher, Waltham, MA, USA) according to the manufacturer’s instructions. For each sample, RNA had previously been isolated from the aqueous phase during the Trizol extraction procedure [13,24], and genomic DNA was then isolated from the remaining organic phase. The concentration of the genomic DNA was determined by Qubit (Thermo Fisher) and the quality of the DNA analyzed by gel electrophoresis. Next generation sequencing of the *TP53* gene was performed using a custom Ion AmpliSeq panel (covering *TP53* and *TP63* genes). Libraries were generated using the Ion AmpliSeq Library Kit 2.0 (Thermo Fisher) according to the manufacturer’s specifications, with 50 ng of genomic DNA as input. Two libraries were prepared with 48 samples in each. The libraries were purified using AMPure XP beads (Agencourt Bioscience, Beverly, MA, USA) and then quantified using the IonLibrary TaqMan Quantitation kit (Thermo Fisher). Sequencing was performed on the Ion Torrent Proton machine (Thermo Fisher) running 48 samples on two Ion P1 chips (Thermo Fisher) according to the manufacturer’s instructions. Data analysis, including alignment to the hg19 human reference genome and mutation calling, was performed in the Ion Reporter Software v.5.4 (Thermo Fisher). The mutation allele frequency cut-off was set at >10%, without adjusting for tumor cellularity. All mutations were confirmed manually using the Integrative Genomics Viewer [38].

### 4.3. Digital PCR Assays

RNA was isolated from fresh frozen breast tumors using TRIzol reagent (Thermo Fisher) and cDNA synthesis performed with 1.5 μg RNA in 20μL reaction volume using SuperScript IV (Thermo Fisher) both as previously described [13]. Long amplicon ddPCR assays were used to quantitate *LRG_321t1*-*t7 TP53* transcripts in breast tumors (Table S4) as described in [13]. The *TP53* 5′ and 3′ end ddPCR assays were performed as previously described [12].

#### 4.3.1. Long Amplicon ddPCR Methodology

For each sample, a 22 μL reaction mix was prepared consisting of 11 μL 2× ddPCR Supermix for Probes (no dUTP) (Bio-Rad, Hercules, CA, USA), 1 μL 20 μM Forward primer, 1 μL 20 μM Reverse primer and 0.28 μL each 20 μM probe (Table 4 and Table 6). DNase/RNase-free water was added to a final volume of 17 μL. To this, 5 μL cDNA diluted in DNase/RNase-free water was added. The droplets were generated and PCR performed as previously described [13], with cycling conditions for each assay described below. Following PCR, the droplets from each well were read on the QX200 droplet reader (Bio-Rad) and data analyzed with QuantaSoft Analysis Pro Software (version 1.0.596) according to manufacturer’s instructions. The threshold was set manually to discriminate between negative and positive droplets. cDNA was substituted with RNase-free water as a non-template control for each probe set, to allow for gating of negative droplets for analysis. The data was visualised in 1-D and 2-D plots using the QuantaSoft Analysis Pro Software [39]. Data from any wells with <10,000 droplets were discarded. Quantitated transcripts were calculated to give the concentration as copies/μg of RNA.

#### 4.3.2. Long Amplicon ddPCR Assays to Detect *TP53* RNAs Expressed in Tumors with *TP53* Gene Splice Site Mutations

Three assays were designed to detect the *TP53* transcripts expressed in the tumors that had splice site mutations reported in the *TP53* gene sequence. These were splice site mutations that were in intron 4, intron 5 and intron 7. The ddPCR assays were set up as described above, using 5 μL cDNA diluted 1:20 in DNase/RNase-free water, or 2ng genomic DNA as a control. Primers and probes for each assay are shown in Table 4 and ddPCR cycling conditions in Table 5.

#### 4.3.3. Long Amplicon Multiplex ddPCR Assay to Quantitate the *TP53* *t8* Transcripts

A long amplicon ddPCR assay was designed to quantitate the *TP53 LRG_321t8* transcript (NCBI transcript identifier NM_001126118.1) encoding ∆40p53α, and also any β or γ versions of this (called *t8β* and *t8γ*, respectively). The ddPCR assay was set up as described above, using 5 μL cDNA diluted 1:10 in DNase/RNase-free water. The assay primers and probes sequences are shown in Table 6.

ddPCR cycling conditions were 94 °C for 10 min, then 50 cycles of 94 °C for 30 s, 64 °C for 1 min, 72 °C for 6 min, followed by 98 °C for 10 min and then hold temperature of 12 °C. The data was quantitated from analysis of 2-D plots (Table S4). As two probes were run in the FAM channel to allow concurrent quantitation of *t8β* and *t8γ* transcripts, analysis was performed using the Advanced Classification Method in the QuantaSoft Analysis Pro Software [39].

### 4.4. Bioinformatics and Statistical Analysis

TCGA BRCA gene expression level 3 data was downloaded on 06/10/2019 [20,21] with *TP53* mutation information downloaded from UCSC Xena [40] on 23042020. Data was plotted using either R or GraphPad Prism8. Nominal variable association of *TP53* transcript information with standard clinical and pathological features was performed using either ANOVA (for breast cancer subtype and histological grade), *t*-test (for tumor ER or PgR status, or patient LN status) or correlation analysis (for patient age at diagnosis). The association of tumor *TP53* information (*TP53* mutation status, the individual *TP53* transcripts or the *TP53* 5′ or 3′ ends) with the time to a distant metastatic event was determined using Cox proportional hazards regression models [41,42]. Note that samples with any missing clinical, pathological or *TP53* information (*n* = 4) were omitted from this analysis. *TP53* information that was a significant prognostic factor in univariable analysis was included with the potential clinical and pathological confounders in a multivariable Cox proportional hazards regression model. All statistical tests were 2-sided, and significance was defined as an alpha of 0.05. Analyses were performed in the R environment [43].

## 5. Conclusions

We have performed an integrated analysis of the *TP53* locus in a New Zealand breast cancer cohort, by sequencing both the *TP53* gene and quantitating the individual *TP53* transcripts expressed from the locus, and combining this with patient clinical and pathological information. Novel multiplex long amplicon ddPCR assay were devised to detect the eighth *TP53* reference transcript, *LRG_321t8*, encoding the ∆40p53α isoform, and also transcripts encoding β and γ versions of this isoform. In the cohort analyzed here, we observed that 16% of *TP53* mutations affect splicing to generate additional *TP53* transcripts, as confirmed in long amplicon ddPCR assays. Those mutations that influenced *TP53* intron 4 splicing were associated with overexpression of transcripts encoding the ∆*133p53* isoforms. We show that incorporating *TP53* transcript information in addition to *TP53* mutation status may be a more accurate predictor of breast cancer patient prognosis.

## Figures and Tables

**Table 1:** Description and frequency of *TP53* mutations in New Zealand breast cancer cohort.

*TP53* Mutation Type	Frequency	Number of Tumors
Frameshift	26%	8
Missense	45%	14
Nonsense	10%	3
Splicing	16%	5
In-Frame indel	3%	1

**Table 2:** Cox univariable analysis of *TP53* tumor information showing association with distant metastases free survival (DMFS) of patients with breast cancer.

*TP53* Information	Raw *p* Value	Adjusted *p* Value ^1^	Hazard Ratio ^2^	95% Confidence Intervals ^2^	Raw *p* Value ^2^	Adjusted *p* Value ^1,2^
*TP53* mutant	2.9 × 10^−3^	0.037	4.36	1.47–12.97	8.1 × 10^−3^	9.2 × 10^−3^
*t2*/*t1* ratio	7.3 × 10^−5^	1.8 × 10^−3^	1.85	1.32–2.59	3.4 × 10^−4^	5.1 × 10^−4^

^1^ False discovery rate (fdr)-adjusted *p* values. ^2^ Adjusted for patient lymph node status, tumor size, estrogen receptor and progesterone receptor status and histological grade.

**Table 3:** Cox multivariable analysis of *TP53* tumor information showing association with DMFS of patients with breast cancer.

*TP53* Information	Hazard Ratio	95% Confidence Intervals	*p* Value
*TP53* mutation	4.61	1.94–10.95	5.4 × 10^−4^
*t2*/*t1* ratio	1.65	1.18–2.32	3.6 × 10^−3^
Tumor size	1.84	1.30–2.61	6.3 × 10^−4^
Lymph node status	4.18	1.50–11.64	6.2 × 10^−3^

**Table 4:** Description of primers and probes for *TP53* splice mutation long amplicon ddPCR assays.

Assay (Intron)	Oligonucleotide	*TP53* Location	Sequence (5′–3′)
4	Forward primer ^1^	Exon 3	ACTTCCTGAAAACAACGTTCTG
4	Reverse primer ^1^	Exon 6	CCACACGCAAATTTCCTTCC
4	Probe_HEX ^1^	Exon 4	5HEX_TGCCCTGGTAGGTTTTCTGGGAAGGGAC_3IABkFQ
5	Forward primer ^2^	Exon 5	CAGCTGTGGGTTGATTCCA
5	Reverse primer ^2^	Exon 7	GTGATGATGGTGAGGATGGG
5	Probe_HEX ^2^	Exon 5	5HEX_TGCTTGTAGATGGCCATGGC_3IABkFQ
7	Forward primer ^3^	Exon 7	CCCATCCTCACCATCATCAC
7	Reverse primer ^3^	Exon 8	GTGAGGCTCCCCTTTCTTG
7	Probe_HEX ^3^	Exon 8	5HEX_ATTCTCTTCCTCTGTGCGCC_3IABkFQ

For assay to detect *TP53* RNAs expressed in tumors with the following splice site mutations; ^1^ Intron 4, ^2^ Intron 5, ^3^ Intron 7.

**Table 5:** Long amplicon ddPCR cycling conditions for the *TP53* splice mutation assays.

Assay	ddPCR Cycling Conditions
Intron 4 splice mutation	94 °C for 10 min, 50 cycles of 94 °C for 30 s, 64 °C for 1 min, 72 °C for 6 min, 98 °C for 10 min and then 12 °C hold
Intron 5 splice mutation	94 °C for 10 min, 50 cycles of 94 °C for 30 s, 62 °C for 30 s, 72 °C for 1 min, 98 °C for 10 min and then 12 °C hold
Intron 7 splice mutation	94 °C for 10 min, 50 cycles of 94 °C for 30 s, 62 °C for 1 min, 72 °C for 3 min, 98 °C for 10 min and then 12 °C hold

**Table 6:** Description of primers and probes for *TP53* *t8*, *t8β* and *t8γ* long amplicon ddPCR assay.

Oligonucleotide	Location	Sequence (5′–3′)
Forward primer	Intron 2	AGTGGATCCATTGGAAGGGCAGGC
Reverse primer ^1^	Exon 10	CTGGGCATCCTTGAGTTCC
α probe_HEX ^1^	Exons 9/10	5HEX_CGGATCTGAAGGGTGAAATATTCTCCA_3IABkFQ
β probe_FAM_1 ^1^	Exon 9β	56-FAM_ACTTTGCCTGATACAGATGCTACT_3IABkFQ
β probe_FAM_2 ^1^	Exon 9β	56-FAM_TCTGTATCAGGCAAAGTCATAGAACCAT_3IABkFQ
γ probe_FAM ^1^	Exons 9/9γ	56-FAM_AGCATCTGAAGGGTGAAATATTCTCCA_3IABkFQ

^1^ Reverse primer and probes the same as *TP53* *t1*/*t3*/*t4* and *t5*/*t6*/*t7* multiplex long amplicon ddPCR assays [13].

## Data Availability

The data presented in this study are available in Table S1, Table S3, Table S4 and [24].

## Supplementary Materials

The following are available online at https://www.mdpi.com/article/10.3390/cancers13071531/s1, Figure S1: Analysis of transcripts encoding ∆40p53 isoforms, Figure S2: *TP53* transcript levels by breast cancer PAM50 subtype, Figure S3: Association of *TP53* mutation status and *TP53* *t3* or *t4* transcript levels with breast cancer patient prognosis, Table S1: Description of *TP53* gene mutations and loss of the wild type *TP53* allele in New Zealand breast tumor cohort, Table S2: Association of *TP53* tumor information with clinical, pathological and intrinsic subtype information in New Zealand breast tumor cohort, Table S3: Clinical and pathological features of 89 breast cancer patients in this study, Table S4: Results from long amplicon ddPCR assays to quantitate 10 *TP53* transcripts in breast cancer samples.

## References

[1] Kandoth C., McLellan M.D., Vandin F., Ye K., Niu B., Lu C., Xie M., Zhang Q., McMichael J.F., Wyczalkowski M.A. (2013). Mutational landscape and significance across 12 major cancer types. Nature.

[2] Bouaoun L., Sonkin D., Ardin M., Hollstein M., Byrnes G., Zavadil J., Olivier M. (2016). TP53Variations in Human Cancers: New Lessons from the IARC TP53 Database and Genomics Data. Hum. Mutat..

[3] Pereira B., Chin S.-F., Rueda O.M., Vollan H.-K.M., Provenzano E., Bardwell H.A., Pugh M., Jones L.A., Russell R., Sammut S.-J. (2016). The somatic mutation profiles of 2433 breast cancers refine their genomic and transcriptomic landscapes. Nat. Commun..

[4] Donehower L.A., Soussi T., Korkut A., Liu Y., Schultz A., Cardenas M., Li X., Babur O., Hsu T.K., Lichtarge O. (2019). Integrated Analysis of TP53 Gene and Pathway Alterations in The Cancer Genome Atlas. Cell Rep..

[5] Banerji S., Cibulskis K., Rangel-Escareno C., Brown K.K., Carter S.L., Frederick A.M., Lawrence M.S., Sivachenko A.Y., Sougnez C., Zou L. (2012). Sequence analysis of mutations and translocations across breast cancer subtypes. Nature.

[6] Silwal-Pandit L., Vollan H.K.M., Chin S.-F., Rueda O.M., McKinney S., Osako T., Quigley D.A., Kristensen V.N., Aparicio S., Børresen-Dale A.-L. (2014). TP53 Mutation Spectrum in Breast Cancer Is Subtype Specific and Has Distinct Prognostic Relevance. Clin. Cancer Res..

[7] Mehta S.Y., Morten B.C., Antony J., Henderson L., Lasham A., Campbell H., Cunliffe H., Horsfield J.A., Reddel R.R., Avery-Kiejda K.A. (2018). Regulation of the interferon-gamma (IFN-gamma) pathway by p63 and Delta133p53 isoform in different breast cancer subtypes. Oncotarget.

[8] Joruiz S.M., Bourdon J.-C. (2016). p53 Isoforms: Key Regulators of the Cell Fate Decision. Cold Spring Harb. Perspect. Med..

[9] Zerbino D.R., Achuthan P., Akanni W., Amode M.R., Barrell D., Bhai J., Billis K., Cummins C., Gall A., Girón C.G. (2018). Ensembl 2018. Nucleic Acids Res..

[10] Dalgleish R., Flicek P., Cunningham F., Astashyn A., Tully R.E., Proctor G., Chen Y., McLaren W.M., Larsson P., Vaughan B.W. (2010). Locus Reference Genomic sequences: An improved basis for describing human DNA variants. Genome Med..

[11] Anbarasan T., Bourdon J.-C. (2019). The Emerging Landscape of p53 Isoforms in Physiology, Cancer and Degenerative Diseases. Int. J. Mol. Sci..

[12] Mehta S., Tsai P., Lasham A., Campbell H., Reddel R., Braithwaite A., Print C. (2016). A Study of TP53 RNA Splicing Illustrates Pitfalls of RNA-seq Methodology. Cancer Res..

[13] Lasham A., Tsai P., Fitzgerald S.J., Mehta S.Y., Knowlton N.S., Braithwaite A.W., Print C.G. (2020). Accessing a New Dimension in TP53 Biology: Multiplex Long Amplicon Digital PCR to Specifically Detect and Quantitate Individual TP53 Transcripts. Cancers.

[14] Avery-Kiejda K.A., Morten B., Wong-Brown M.W., Mathe A., Scott R.J. (2013). The relative mRNA expression of p53 isoforms in breast cancer is associated with clinical features and outcome. Carcinogenesis.

[15] Bourdon J.-C., Khoury M.P., Diot A., Baker L., Fernandes K., Aoubala M., Quinlan P., Purdie A.C., Jordan L.B., Prats A.-C. (2011). p53 mutant breast cancer patients expressing p53γ have as good a prognosis as wild-type p53 breast cancer patients. Breast Cancer Res..

[16] Gadea G., Arsic N., Fernandes K., Diot A., Joruiz S.M., Abdallah S., Meuray V., Vinot S., Anguille C., Remenyi J. (2016). Tp53 drives invasion through expression of its Δ133p53β variant. eLife.

[17] Razavi P., Chang M.T., Xu G., Bandlamudi C., Ross D.S., Vasan N., Cai Y., Bielski C.M., Donoghue M.T., Jonsson P. (2018). The Genomic Landscape of Endocrine-Resistant Advanced Breast Cancers. Cancer Cell.

[18] Curtis C., Shah S.P., Chin S.F., Turashvili G., Rueda O.M., Dunning M.J., Speed D., Lynch A.G., Samarajiwa S., Yuan Y. (2012). The genomic and transcriptomic architecture of 2000 breast tumours reveals novel subgroups. Natature.

[19] Leroy B., Anderson M., Soussi T. (2014). TP53 Mutations in Human Cancer: Database Reassessment and Prospects for the Next Decade. Hum. Mutat..

[20] Grossman R.L., Heath A.P., Ferretti V., Varmus H.E., Lowy D.R., Kibbe W.A., Staudt L.M. (2016). Toward a Shared Vision for Cancer Genomic Data. N. Engl. J. Med..

[21] TCGA BRCA Gene Expression Level 3 Data. https://gdac.broadinstitute.org/.

[22] Ghosh A., Stewart D., Matlashewski G. (2004). Regulation of Human p53 Activity and Cell Localization by Alternative Splicing. Mol. Cell. Biol..

[23] Sorlie T., Tibshirani R., Parker J., Hastie T., Marron J.S., Nobel A., Deng S., Johnsen H., Pesich R., Geisler S. (2003). Repeated observation of breast tumor subtypes in independent gene expression data sets. Proc. Natl. Acad. Sci. USA.

[24] Muthukaruppan A., Lasham A., Blenkiron C., Woad K.J., Black M.A., Knowlton N., McCarthy N., Findlay M.P. (2017). Genomic profiling of breast tumours from New Zealand patients. N. Z. Med. J..

[25] Haybittle J.L., Blamey R.W., Elston C.W., Johnson E.J., Doyle P.J., Campbell F.C., Nicholson I.R., Griffiths K. (1982). A prognostic index in primary breast cancer. Br. J. Cancer.

[26] Ravdin P.M., Siminoff L.A., Davis G.J., Mercer M.B., Hewlett J., Gerson N., Parker H.L. (2001). Computer Program to Assist in Making Decisions About Adjuvant Therapy for Women with Early Breast Cancer. J. Clin. Oncol..

[27] Wishart G.C., Azzato E.M., Greenberg D.C., Rashbass J., Kearins O., Lawrence G., Caldas C., Pharoah P.D. (2010). Predict: A new UK prognostic model that predicts survival following surgery for invasive breast cancer. Breast Cancer Res. BCR.

[28] Calabrese C., Davidson N.R., Demircioglu D., Fonseca N.A., He Y., Kahles A., Lehmann K.V., Liu F., Shiraishi Y., Soulette C.M. (2020). Genomic basis for RNA alterations in cancer. Nat. Cell Biol..

[29] Olivier M., Bouaoun L., Sonkin D., Ardin M., Hollstein M., Byrnes G., Zavadil J. (2016). TP53 variations in human cancers: New lessons from the IARC TP53 Database and genomic studies. Eur. J. Cancer.

[30] Smeby J., Sveen A., Eilertsen I.A., Danielsen S.A., Hoff A.M., Eide P.W., Johannessen B., Hektoen M., Skotheim R.I., Guren M.G. (2019). Transcriptional and functional consequences of TP53 splice mutations in colorectal cancer. Oncogenesis.

[31] Jayasinghe R.G., Cao S., Gao Q., Wendl M.C., Vo N.S., Reynolds S.M., Zhao Y., Climente-González H., Chai S., Wang F. (2018). Systematic Analysis of Splice-Site-Creating Mutations in Cancer. Cell Rep..

[32] Soukarieh O., Gaildrat P., Hamiet M., Drouet A., Baert-Desurmont S., Frébourg T., Tosi M., Martins A. (2016). Exonic splicing mutations are more prevalent than currently estimated and can be predicted using in silico tools. PLoS Genet..

[33] Garziera M., Cecchin E., Giorda G., Sorio R., Scalone S., De Mattia E., Roncato R., Gagno S., Poletto E., Romanato L. (2019). Clonal Evolution of TP53 c.375 + 1G > A Mutation in Pre-and Post-Neo-Adjuvant Chemotherapy (NACT) Tumor Samples in High-Grade Serous Ovarian Cancer (HGSOC). Cells.

[34] Frebourg T., Barbier N., Yan Y.X., Garber E.J., Dreyfus M., Fraumeni J., Li F.P., Friend S.H. (1995). Germ-line p53 mutations in 15 families with Li-Fraumeni syndrome. Am. J. Hum. Genet..

[35] Koster J., Plasterk R.H.A. (2019). A library of Neo Open Reading Frame peptides (NOPs) as a sustainable resource of common neoantigens in up to 50% of cancer patients. Sci. Rep..

[36] Duffy M.J., Synnott N.C., Crown J. (2018). Mutant p53 in breast cancer: Potential as a therapeutic target and biomarker. Breast Cancer Res. Treat..

[37] Statistics New Zealand Ethnic Group Summaries Reveal New Zealand’s Multicultural Make-Up. https://www.stats.govt.nz/news/ethnic-group-summaries-reveal-new-zealands-multicultural-make-up.

[38] Thorvaldsdóttir H., Robinson J.T., Mesirov J.P. (2012). Integrative Genomics Viewer (IGV): High-performance genomics data visualization and exploration. Brief. Bioinform..

[39] Droplet Digital PCR. Laboratories. B.-R. Applications Guide. https://www.bio-rad.com/webroot/web/pdf/lsr/literature/Bulletin_6407.pdf.

[40] Goldman M.J., Craft B., Hastie M., Repečka K., McDade F., Kamath A., Banerjee A., Luo Y., Rogers D., Brooks A.N. (2020). Visualizing and interpreting cancer genomics data via the Xena platform. Nat. Biotechnol..

[41] Harrington D., Fleming T. (1982). A class of rank test procedures for censored survival data. Biometrika.

[42] Therneau T. (2011). Survival: Survival Analysis, Including Penalized Likelihood.

[43] R Core Team (2014). R: A Language and Environment for Statistical Computing.

