# Supplementary material for: Breast Cancer Patient Prognosis Is Determined by the Interplay between TP53 Mutation and Alternative Transcript Expression: Insights from TP53 Long Amplicon Digital PCR Assays
Source: Cancers (Basel). 2021 Mar 26;13(7):1531. doi: 10.3390/cancers13071531 (PMC8036703; doi:10.3390/cancers13071531)
Supplement: Supplementary file 1 [file cancers-13-01531-s001.zip › Supplementary data/Supplementary Tables.pdf]

Table S1. Description of *TP53* gene mutations and loss of the wild type *TP53* allele in New Zealand breast tumor cohort

Sample name	*TP53* Mutation	cDNA info	COSMIC ID	Mutation Type	Mutant allele frequency[1]	*TP53* LOH [2]
BC0009R	p.S46*fs*1	c.137_141delCCCCG		Frameshift	20%	Yes
AL0014	p.E51Vfs*4	c.152_156delAACAA		Frameshift	20%	No
AL0088	p.P82Rfs*41	c.245delC		Frameshift	74%	No
AL0027	p.G105V	c.314G>T	COSM46161	Missense	36%	No
AL0011	p.R110P	c.329G>C	COSM11250	Missense	29%	Yes
AL0039	p.R110Pfs*39	c.328dup	COSM96579	Frameshift	29%	No
BC0003	p.N131fs	c.393delTCTTGGCCAGTT GGCAAAACATCTTGTTGA GGGCAGG		Frameshift	13%	No
AL0021	inside intron 4	c.375+1G>A	COSM45304	Splicing	58%	Yes
AL0060	inside intron 4	c.375+5G>A	COSM6222489	Splicing	53%	No
AL0001	p.P151S	c.451C>T	COSM10905	Missense	50%	No
AL0067	p.P151R	c.452C>G	COSM44003	Missense	52%	Yes
BC0043	p.C176Y	c.527G>A	COSM10687	Missense	23%	No
AL0069	p.H179D	c.535C>G	COSM44776	Missense	34%	No
AL0068	inside intron 5	c.560-1G>A	COSM43753	Splicing	13%	No
AL0078	p.R213*	c.637C>T	COSM10654	Nonsense	17%	No
AL0065	p.Y220C	c.659A>G	COSM10758	Missense	23%	Yes
AL0063	p.Y220C	c.659A>G	COSM10758	Missense	46%	No
AL0043	p.Y234fs*4	c.699-700insTGGA		Frameshift	19%	No
BC0010	p.M237I	c.711G>T	COSM11063	Missense	35%	No
AL0057	p.I251N	c.752T>A	COSM11374	Missense	62%	No
AL0007	p.I255delI	c.764_766delTCA	COSM43694	In-Frame Deletion	79%	Yes
AL0062	p.D259V	c.776A>T	COSM43724	Missense	27%	No
AL0034	from exon 7 to intron 7	c.769_782+7delCTGGAA GACTCCAGGTCAGGA		Splicing	32%	No
AL0073	inside intron 7	c.782+1G>A	COSM43571	Splicing	39%	No
BC0006	p.N263Rfs*80	c.788_794delATCTACT		Frameshift	59%	No
BC0020	p.R267W	c.799C>T	COSM11183	Missense	29%	No
BC0019	p.P278A	c.832C>G	COSM10814	Missense	57%	No
AL0029	p.E286K	c.856G>A	COSM10726	Missense	44%	No
AL0013	p.Y327Ffs*9	c.980_981delAT	COSM45832	Frameshift	38%	No
AL0086	p.E339*	c.1015G>T	COSM11286	Nonsense	40%	Yes
AL0042	p.R342*	c.1024C>T	COSM11073	Nonsense	50%	Yes

[1]not adjusted for tumor cellularity, [2]LOH = likely loss of heterozygosity

Table S2. Association of *TP53* tumor information with clinical, pathological and intrinsic subtype information in New Zealand breast tumor cohort. Table shows *p* values, with significant *p* values highlighted in bold.

***TP53* transcript information**	**Breast cancer subtype**	**Tumor ER status**	**Tumor PgR status**	**Histological Grade**	**Patient Age**	**Patient LN status**
t1	0.450	0.958	0.771	0.876	0.858	0.952
t2	0.351	0.925	0.650	0.815	0.604	0.944
t3	0.134	**0.047**	0.077	0.437	0.136	0.491
t4	0.112	**0.032**	**0.037**	0.098	0.516	**0.043**
t5	0.476	0.159	0.159	0.249	0.056	0.170
t6	0.526	0.164	0.165	0.258	**0.049**	0.186
t7	0.404	0.182	0.187	0.277	0.091	0.222
t8	**0.042**	0.750	0.830	0.488	0.289	0.406
t8β	0.447	0.400	0.156	0.133	0.558	0.106
t8γ	0.500	0.599	0.160	0.256	0.251	0.925
t2/t1	0.982	0.558	0.865	0.887	0.074	**0.042**
t3/t1	**9.8×10^{-5}**	**4.7×10^{-4}**	**0.001**	0.276	0.056	0.339
t4/t1	0.231	0.113	0.114	0.173	0.260	0.079
t5/t1	0.489	0.157	0.154	0.244	0.054	0.174
t6/t1	0.513	0.164	0.161	0.265	**0.048**	0.216
t7/t1	0.442	0.176	0.183	0.285	0.084	0.214
t8/t1	0.092	0.970	0.414	0.414	0.081	0.248
t8β/t1	0.416	0.643	0.108	0.194	0.784	0.213
t8γ/t1	0.452	0.700	0.163	0.258	0.215	0.782
FL/Δ40TP53_T1 5′end	0.351	0.417	0.118	0.195	0.091	0.547
FL/Δ40TP53_T2 5′end	0.726	0.851	0.640	0.736	**0.034**	0.520
Δ133TP53 5′ end	0.221	0.082	**0.049**	**0.05**	**0.038**	0.283
TP53α 3′ end	0.770	0.341	0.539	0.796	0.564	0.854
TP53β 3′ end	**0.026**	0.076	0.057	0.170	0.770	0.699
TP53 mutation	**4.7e-5**	**1.3×10^{-4}**	**6.5×10^{-6}**	**4.4×10^{-6}**	0.059	0.653
TP53 LOH	0.920	0.650	0.703	0.470	0.210	0.610

ER= estrogen receptor, PgR= progesterone receptor, LN= Lymph node, LOH = loss of heterozygosity

Table S3. Clinical and pathological features of 89 breast cancer patients in this study; a subset of the cohort described in [24].

Clinicopathological feature	Mean (range)	Number (% of total)
Patient age (years)	60 (31-94)	/
Tumor size (mm)	30 (6-100)	/
Patient lymph node status		
LN+	/	47 (53%)
LN-	/	39 (44%)
N/A	/	3 (3%)
Histological tumor grade		
Grade 1	/	6 (7%)
Grade 2	/	35 (39%)
Grade 3	/	48 (54%)
Tumor ER status		
ER+	/	63 (71%)
ER-	/	26 (29%)
Tumor PgR status		
PgR+	/	52 (58%)
PgR-	/	36 (41%)
N/A	/	1 (1%)
Breast cancer subtype		
Luminal A	/	21 (24%)
Luminal B	/	27 (30%)
Her2+	/	14 (16%)
Basal-like	/	19 (21%)
Normal-like	/	8 (9%)

LN= Lymph node, ER= estrogen receptor, PgR= progesterone receptor

Table S4. Results from long amplicon ddPCR assays to quantitate 10 *TP53* transcripts in breast cancer samples. Transcripts are *LRG_321t1-t8*, and non-reference transcripts we have named *t8β* and *t8γ*. Values shown are the number of copies of each transcript/μg RNA. *TP53* transcripts are predicted to encode the following p53 isoforms; *t1* and *t2* = FL/Δ40p53α, *t3* = FL/Δ40p53β, *t4* = FL/Δ40p53γ, *t5* = Δ133/Δ160p53α, *t6* = Δ133/Δ160p53β, *t7* = Δ133/Δ160p53γ, *t8* = Δ40p53α, *t8β* = Δ40p53β, *t8g* = Δ40p53γ.

Sample name	*t1*	*t2*	*t3*	*t4*	*t5*	*t6*	*t7*	*t8*	*t8β*	*t8γ*
BC0009R	25752	638	654	0	65	0	0	208	35	0
AL0014	184265	1884	1816	0	221	26	0	648	38	0
AL0088	113667	801	1574	79	1000	74	0	1309	0	0
AL0027	1431074	13944	3336	0	5903	423	11	6442	84	0
AL0011	778216	7234	2407	0	3650	404	14	3686	69	0
AL0039	140400	1524	3367	0	1412	210	0	1293	76	0
AL0021	491586	4491	19972	170	448320	33117	232	4150	248	0
AL0060	599823	5255	6007	76	478512	23700	432	1082	0	0
AL0001	735612	5421	819	0	393	25	0	3499	0	0
AL0067	938885	8856	2370	79	1165	117	0	2709	47	0
BC0043	111553	618	507	0	329	12	0	259	0	0
AL0069	1574766	12556	3443	70	1752	73	0	10572	66	0
AL0078	174015	1633	2579	74	450	16	0	1196	39	0
AL0063	629101	5926	764	0	449	120	0	17184	356	0
AL0065	259254	2200	282	0	253	77	0	5979	448	0
AL0043	354623	3654	3037	0	285	36	0	1253	76	0
BC0010	193327	2476	86	0	449	12	0	1184	0	0
AL0057	458532	4185	1653	83	410	23	0	1510	41	0
AL0062	50118	726	85	0	141	12	0	275	0	0
AL0034	307482	2739	1501	79	75	15	0	1382	36	0
AL0007	1088618	10954	6961	158	1169	91	0	8450	152	0
BC0020	25298	496	169	0	14	0	0	70	0	0
BC0019	285164	3171	303	0	138	0	0	2024	0	0
AL0029	703912	7882	1125	0	148	12	0	1553	0	0
AL0013	107760	1323	1532	0	97	36	0	184	0	0
AL0086	545126	4744	3589	0	785	36	0	5873	139	35
AL0042	1002802	8620	6071	89	3745	251	0	17169	714	0
AL0002	510354	4573	2988	0	969	94	0	1903	68	0
AL0003	507144	4139	1229	0	394	0	0	1953	0	0
AL0004	433023	4202	285	0	395	12	0	3116	0	0
AL0005	224411	2198	1288	0	290	29	0	699	39	0
AL0006	374705	2918	82	0	119	12	0	2296	40	0
AL0008	214791	2286	2358	0	388	315	0	1075	72	0
AL0010	361634	3366	1400	0	133	97	0	2324	169	0
AL0012	360056	2830	748	0	564	25	0	4578	114	0
AL0015	593771	5685	3593	78	275	69	0	4725	171	0
AL0017	117985	679	1268	0	1180	141	0	1898	107	0
AL0018	182851	1749	435	0	50	25	0	3044	112	0
AL0019	460994	3649	1143	0	93	0	0	3369	0	0
AL0020	199157	1475	2434	0	267	116	0	1654	81	0
AL0022	319407	3270	834	0	116	0	0	4556	0	0

AL0023	455022	4273	2505	0	167	71	0	4279	34	0
AL0024	297951	2405	1145	163	475	24	0	3895	40	0
AL0025	497381	5029	2734	78	584	40	0	2556	0	0
AL0026	329180	3463	2220	79	445	54	13	2795	36	0
AL0028	365173	3174	2515	93	190	63	0	1532	39	0
AL0030	18346	253	72	0	0	0	0	71	0	0
AL0032	142194	1635	445	0	35	12	0	676	0	0
AL0033	579293	5359	2167	0	1139	96	0	6508	80	0
AL0035	182358	2528	1200	0	63	0	0	1113	0	0
AL0036	948026	8442	2622	0	299	0	0	7435	0	0
AL0037	647280	5026	2446	0	1558	166	0	4149	69	0
AL0040	99904	930	336	0	149	27	0	673	32	0
AL0041	729773	6755	2733	0	329	88	0	5364	34	0
AL0044	274098	2614	5203	0	104	161	0	2528	34	0
AL0046	299481	3093	2102	0	486	58	0	4510	76	0
AL0047	1064689	9268	3692	0	1827	97	0	4516	63	0
AL0048	424828	3261	1796	82	139	35	12	1144	35	0
AL0049	1406952	11926	2376	0	606	23	0	6812	75	0
AL0050	536942	4165	5226	0	661	139	0	867	0	0
AL0051	244428	1986	1894	146	82	23	0	731	0	0
AL0052	415262	3823	4794	0	122	24	0	2423	98	33
AL0053	281597	3264	2475	0	87	12	0	2539	0	0
AL0054	718424	5439	6435	0	436	97	0	3729	0	0
AL0056	169021	1855	1081	0	867	115	0	743	87	0
AL0058	995838	9224	4222	86	432	23	0	5037	119	0
AL0061	612785	5971	2020	75	895	82	0	7221	39	0
AL0070	107826	1119	1838	0	123	61	0	687	0	0
AL0071	629630	6573	1961	0	257	82	0	1750	0	0
AL0072	308586	3364	1478	0	170	24	0	1276	34	0
AL0074	87490	727	324	0	200	0	0	315	0	0
AL0075	431040	3869	1320	0	135	51	0	2700	67	0
AL0077	27516	101	273	0	0	0	0	68	0	0
AL0079	363819	3625	634	0	0	0	0	1997	0	0
AL0087	663337	6170	4987	0	185	12	0	3118	107	0
BC0015	34029	285	394	79	36	12	0	67	0	0
BC0050	272193	3121	190	0	225	0	0	1040	0	0
