# Supplementary material for: Breast Cancer Patient Prognosis Is Determined by the Interplay between TP53 Mutation and Alternative Transcript Expression: Insights from TP53 Long Amplicon Digital PCR Assays
Source: Cancers (Basel). 2021 Mar 26;13(7):1531. doi: 10.3390/cancers13071531 (PMC8036703; doi:10.3390/cancers13071531)
Supplement: Supplementary file 1 [file cancers-13-01531-s001.zip › Supplementary data/Supp_Figures.pdf]

(a)

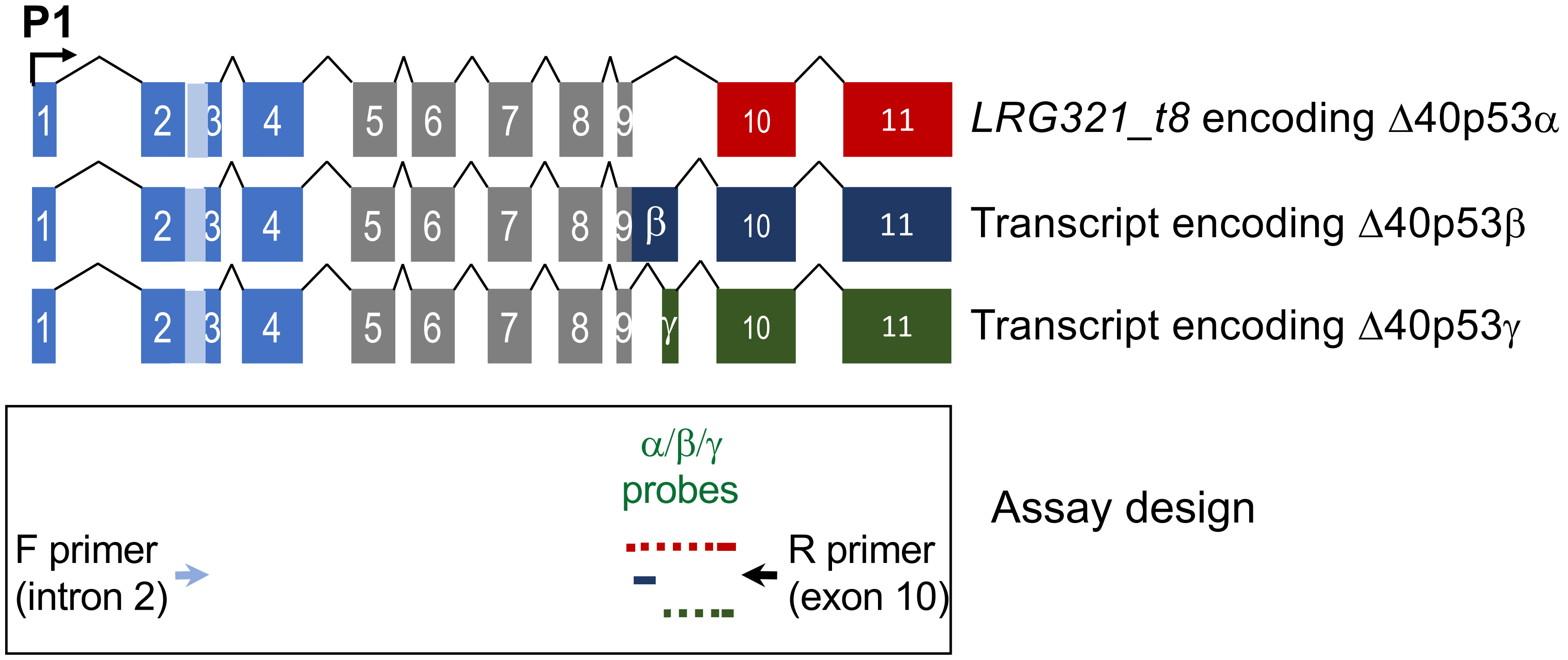

(b)

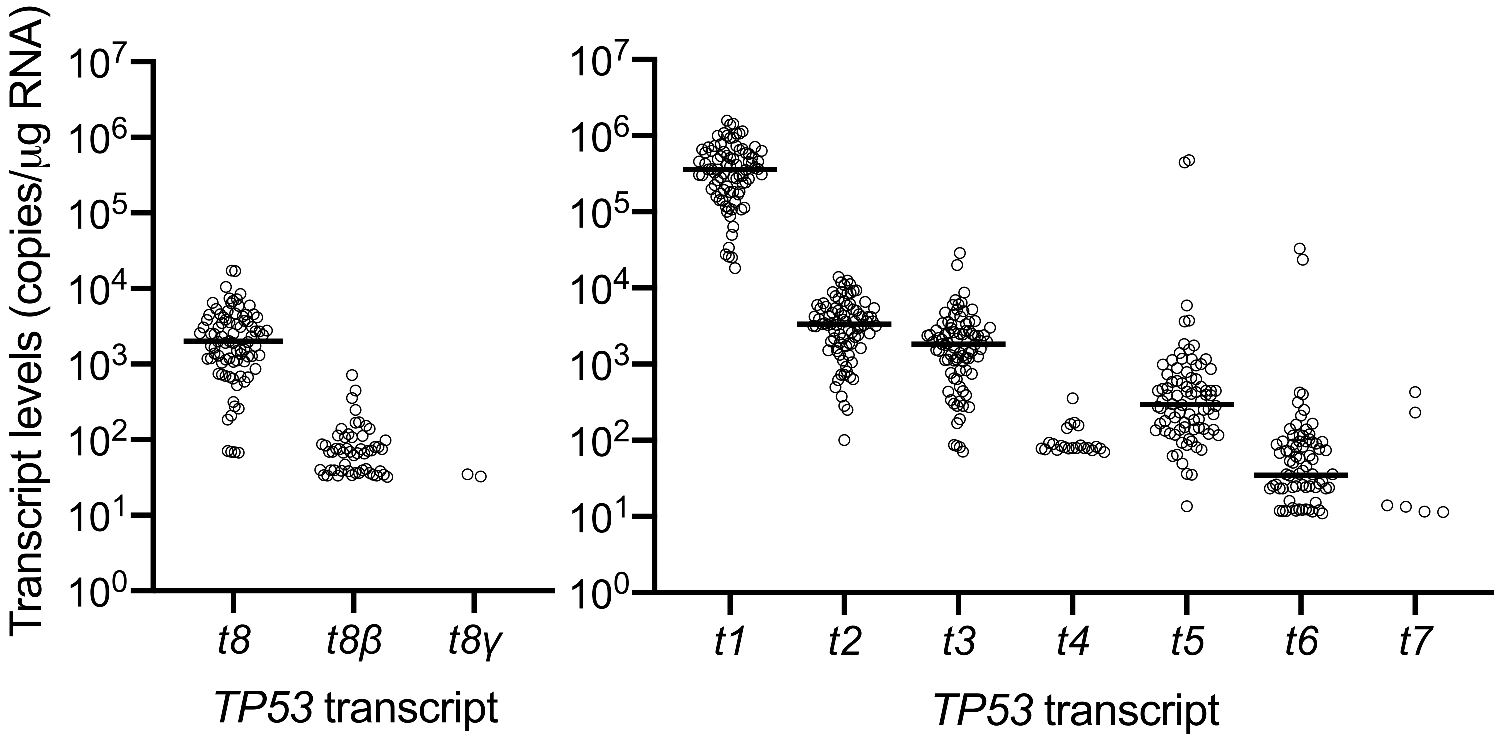

Figure S1. Analysis of transcripts encoding Δ40p53 isoforms. **a**) long amplicon ddPCR assay to detect *TP53* transcript *t8* (encoding Δ40p53α) and β and γ versions of this ("*t8β*" and "*t8γ*" respectively). **b**) levels of *TP53 t8*, *t8β* and *t8γ* transcripts in NZ breast cancer cohort, plotted alongside *t1-t7* transcripts. Circles represent individual tumor samples, with horizontal line showing the median. Note that transcripts that were less than 10 copies/µg RNA were undetectable.

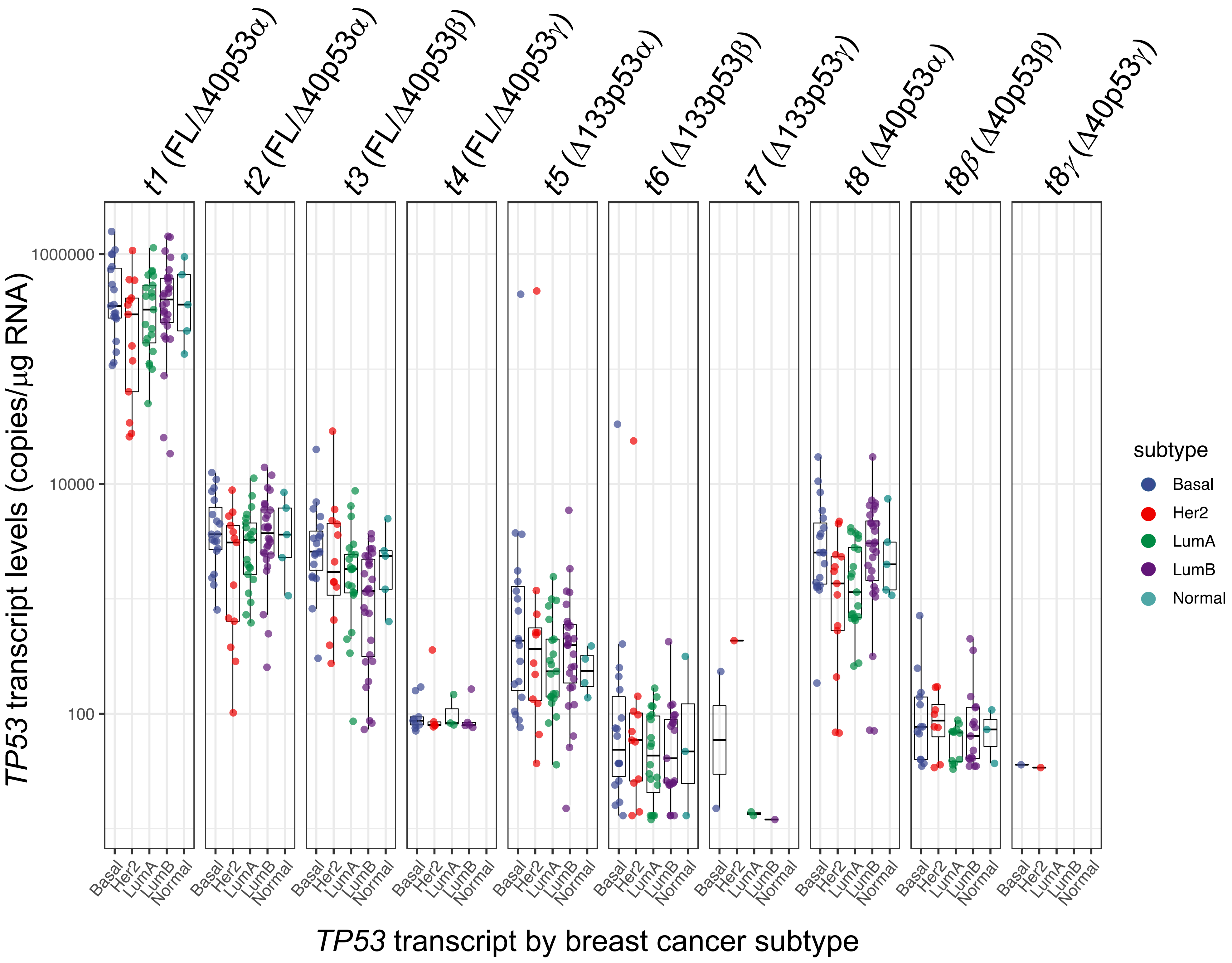

Figure S2. *TP53* transcript levels by breast cancer PAM50 subtype. Box and scatterplots showing the copies/μg tumor RNA of each *TP53* transcript plotted by breast cancer subtype. Colored dots represent individual tumor samples, with boxplot showing the median and 75th to 25th percentiles, with whiskers extending to 1.5x interquartile range. Note that transcripts that were less than 10 copies/μg RNA were undetectable. Basal = Basal-like (n=19), Her2 = HER2-enriched (n=13) , LumA= Luminal A (n=21), LumB= Luminal B (n=27), Normal= Normal-like (n=5).

Figure S3

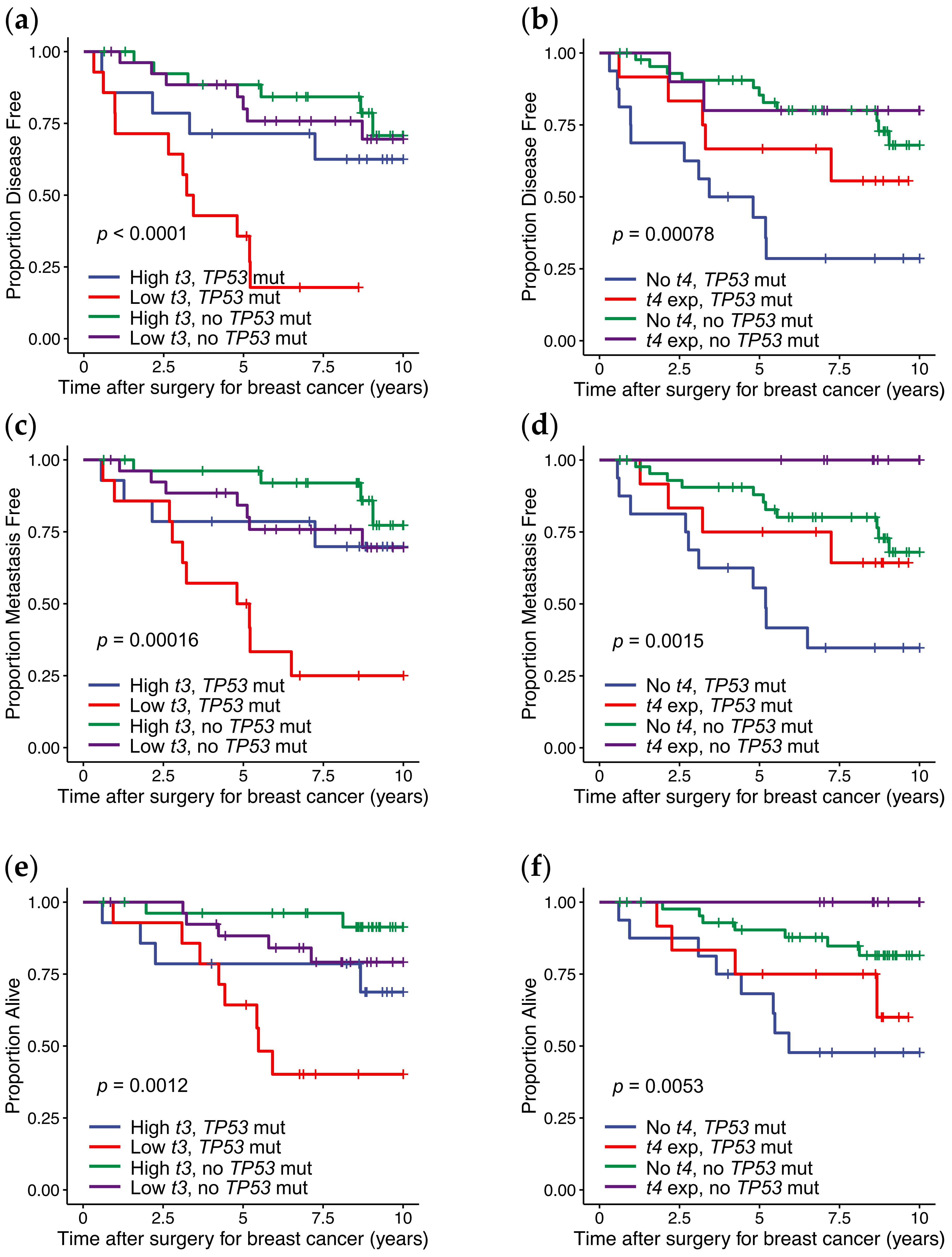
(a)
Proportion Disease Free
p < 0.0001
High t3, TP53 mut
Low t3, TP53 mut
High t3, no TP53 mut
Low t3, no TP53 mut
Time after surgery for breast cancer (years)
(b)
Proportion Disease Free
p = 0.00078
No t4, TP53 mut
t4 exp, TP53 mut
No t4, no TP53 mut
t4 exp, no TP53 mut
Time after surgery for breast cancer (years)
(c)
Proportion Metastasis Free
p = 0.00016
High t3, TP53 mut
Low t3, TP53 mut
High t3, no TP53 mut
Low t3, no TP53 mut
Time after surgery for breast cancer (years)
(d)
Proportion Metastasis Free
p = 0.0015
No t4, TP53 mut
t4 exp, TP53 mut
No t4, no TP53 mut
t4 exp, no TP53 mut
Time after surgery for breast cancer (years)
(e)
Proportion Alive
p = 0.0012
High t3, TP53 mut
Low t3, TP53 mut
High t3, no TP53 mut
Low t3, no TP53 mut
Time after surgery for breast cancer (years)
(f)
Proportion Alive
p = 0.0053
No t4, TP53 mut
t4 exp, TP53 mut
No t4, no TP53 mut
t4 exp, no TP53 mut
Time after surgery for breast cancer (years)

Figure S3. Association of *TP53* mutation status and *t3* or *t4* transcript levels with breast cancer patient prognosis. Kaplan-Meier curves showing the proportion of patients having any breast cancer recurrence (**a**, **b**), having a distant metastatic event (**c**, **d**) or dying from breast cancer (**e**, **f**). **a**), **c**), **e**) *TP53* tumor mutation status and *t3* levels are used to stratify patients into four groups "High" = greater than median and "Low" = less than median levels of *t3*, Blue line = patients with tumors with High *t3* levels and *TP53* mutation (n = 14), Red line = patients with tumors with Low t3 levels and *TP53* mutation (n = 14), Green line = patients with tumors with High *t3* levels and no *TP53* mutation (n = 28), Purple line = patients with tumors with Low *t3* levels and no *TP53* mutation (n = 27). **b**), **d**), **f**) *TP53* tumor mutation status and *t4* levels are used to stratify patients into four groups. "No" = undetectable levels of *t4* and "exp" = detectable levels of *t4* transcript, Blue line = patients with tumors with undetectable *t4* and *TP53* mutation (n = 16), Red line = patients with tumors with detectable levels of *t4* and *TP53* mutation (n = 12), Green line = patients with tumors with undetectable *t4* levels and no *TP53* mutation (n = 45), Purple line = patients with tumors with detectable *t4* levels and no *TP53* mutation (n = 10). Log rank test *p* values are shown on each graph.
